# Pragmatic, feasibility randomized controlled trial of a recorded mental health recovery narrative intervention: narrative experiences online intervention for informal carers (NEON-C)

**DOI:** 10.3389/fpsyt.2023.1272396

**Published:** 2024-01-23

**Authors:** Fiona Ng, Stefan Rennick-Egglestone, Juliana Onwumere, Christopher Newby, Joy Llewellyn-Beardsley, Caroline Yeo, Yasmin Ali, Kristian Pollock, Yasuhiro Kotera, Scott Pomberth, Sean P. Gavan, Lian van der Krieke, Dan Robotham, Steve Gillard, Graham Thornicroft, Mike Slade, and the NEON Study Group

**Affiliations:** ^1^School of Health Sciences, Institute of Mental Health, University of Nottingham, Nottingham, United Kingdom; ^2^Department of Psychology, Institute of Psychiatry, Psychology and Neuroscience, King's College London, London, United Kingdom; ^3^South London and Maudsley NHS Foundation Trust, Bethlem Royal Hospital, Kent, United Kingdom; ^4^School of Health Sciences, University of Nottingham, Nottingham, United Kingdom; ^5^NEON Lived Experience Advisory Panel, Nottingham, United Kingdom; ^6^Manchester Centre for Health Economics, Division of Population Health, Health Services Research and Primary Care, The University of Manchester, Manchester, United Kingdom; ^7^University Medical Center Groningen, University Center of Psychiatry, University of Groningen, Groningen, Netherlands; ^8^McPin Foundation, London, United Kingdom; ^9^School of Health & Psychological Sciences, City, University of London, London, United Kingdom; ^10^Centre for Implementation Science, Health Service and Population Research Department, Institute of Psychiatry, Psychology and Neuroscience, King's College London, London, United Kingdom; ^11^Health and Community Participation Division, Faculty of Nursing and Health Sciences, Nord University, Namsos, Norway

**Keywords:** mental health, recovery, recovery narratives, carers, NEON intervention, recommender system, digital health intervention, online intervention

## Abstract

**Introduction:**

Informal carers of people with mental health problems often have unmet support needs. Mental health recovery narratives are increasingly accessible, but their relevance to and effect on informal carers have been minimally investigated. The Narrative Experiences Online (NEON) Intervention is a first-in-field intervention that provides informal carers with access to a diverse collection of recorded mental health recovery narratives. This trial aimed to examine the feasibility and acceptability of the NEON Intervention for informal carers.

**Methods:**

This study involved a two-arm feasibility randomized controlled trial. Carers were randomly assigned to receiving versus not receiving the NEON Intervention. The feasibility aspects investigated included the acceptability of the intervention and of randomization, trial processes, engagement rates, recruitment procedures, attrition, sample size estimation, identification of candidate primary and secondary outcomes, and the feasibility of conducting a definitive trial. A qualitative process evaluation was conducted.

**Findings:**

A total of 121 carers were eligible, of whom 54 were randomized (intervention: 27, control: 27). Twelve-month follow-up data were available for 36 carers. Carers accessed a mean of 25 narratives over a 12-month period, and the intervention group, compared with the control group, reported a small effect on hope and a moderate effect on the presence of meaning in life. Five modifications were recommended to improve the user experience, applicability, and trial processes.

**Discussion:**

The NEON Intervention is feasible and acceptable. Significant refinement of the NEON Intervention and trial processes is required to personalize and ensure applicability to carers. Further feasibility testing is recommended prior to a definitive trial.

## 1 Introduction

Informal carers of people with mental health problems, henceforth called carers in this study, are commonly described as ‘someone who provides unpaid help to a friend or family member needing support’ (1). Carers provide emotional or practical support, for example, helping to identify when initial distress or relapse arises, advocating, and supporting self-management (2). Caring can be challenging, with carers experiencing poorer mental health and lower positive wellbeing compared to non-carers (3).

Digital interventions to support carers’ mental health and wellbeing have been developed and evaluated (4, 5). These can assist in overcoming caring challenges by improving access to evidence-based support while allowing engagement flexibility. One under-researched approach is the use of narratives, which describe recovery from mental health problems, as a resource for carers. Mental health recovery narratives are defined as first-person lived experience accounts, including elements of success and adversity that are, in part, related to mental health problems (6). Mental health recovery narratives are available in the public domain (for example, through books, written collections, YouTube videos, and blogs) and are a feature within clinical and public health interventions (for example, peer support and anti-stigma campaigns) (7, 8). Utilizing mental health recovery narratives as a resource to support carers has warranted limited empirical investigation. We previously developed a digital intervention that provides people with mental health problems access to recovery narratives, called the NEON Intervention (9). This study evaluated the feasibility of the NEON Intervention for informal carers of people with mental health problems. Findings will inform the design of a future definitive randomized control trial (RCT). The objectives were as follows:

Objective 1 (trial parameters): To evaluate the feasibility of conducting a future definitive trial by evaluating recruitment procedures, participant attrition rates, recruitment rates, sample size estimates, and primary and secondary outcome identification of candidates.

Objective 2 (acceptability): To investigate participant satisfaction with the NEON Intervention (including randomization acceptability and engagement rates).

Objective 3 (feasibility): To evaluate the suitability of the NEON Intervention and of trial processes for use by informal carers of people with mental health problems.

## 2 Materials and methods

The NEON Intervention was evaluated within three concurrent trials targeted at different population groups, comprising people with the experience of psychosis (NEON; ISRCTN11152837), people with mental health concerns other than psychosis (NEON-O; ISRCTN63197153), both sets of people but reported elsewhere, and carers (NEON-C; ISRCTN76355273) (10, 11). The trials were conducted and reported in line with the Consolidated Standards of Reporting Trials (CONSORT) checklist for feasibility and pilot trials (12).

### 2.1 Design

The design was an online, individually, randomized, feasibility RCT of the NEON Intervention for carers of people with mental health problems, compared with a one-year waitlist control, with embedded process evaluation. All participants continued with any support for their carer role during the trial and were followed up for 52 weeks. The study received ethical approval (East Midlands Leicester REC 19/EM/0326). The trial was overseen by a Program Steering Committee (PSC), a Trial Management Group (TMG), and a Lived Experience Advisory Panel (LEAP).

### 2.2 Participants and recruitment

Participant inclusion was assessed through an online eligibility checking interface shared between the three NEON trials (10, 11). The NEON-C inclusion criteria were as follows: participants aged 18 years or older, those with self-reported experience of caring (defined as unpaid care) for someone with mental health problems (diagnosed/self-reported) in the past 5 years, those who live in England, those who are capable of accessing or who are being supported to access the Internet, either on a personal computer, mobile device, or at a community venue, those who are able to understand written or spoken English, and those who are able to provide online informed consent. The exclusion criteria were as follows: self-reported experience of psychosis or other mental health problems in the past 5 years and mental health-related distress in the past 6 months, which met inclusion in the NEON or NEON-O trials.

A recruitment strategy was implemented to recruit carers through multiple routes, including advertising in print media, snowball recruitment, 11 NHS mental health trusts in England, health professionals (e.g., general practitioners and psychiatrists), and national and social media. Recruitment materials followed a pre-defined set of principles to provide ethical oversight and to ensure consistency (13).

### 2.3 Intervention

The NEON Intervention is a novel first-in-field intervention that facilitates online access to recorded mental health recovery narratives. Developed in collaboration with a Lived Experience Advisory Panel (LEAP), the NEON Intervention is fully online with no face-to-face contact with researchers or clinicians. It provided free access to a curated and diverse (14) collection of recorded mental health recovery narratives (NEON Collection) (9), which consisted of 687 diverse lived experience narratives from people with mental health problems.

Foundational research examining the impact of mental health recovery narratives on people with mental health problems has been conducted. The Narrative Experiences Online (NEON) Impact Model developed using systematic reviews (15), interview analyses (16), and experimental methods (17, 18) describes the moderators, mechanisms, and processes by which engaging with a recorded mental health recovery narrative may lead to outcomes (9). Following engagement with a recovery narrative, individuals reflect upon their own experiences, and a connection with the narratives can occur through one of the three mechanisms: comparison, learning, or empathy. Processes by which connection leads to outcome include the identification of change through either the narrative structure or content, leading to the internalization of the recipient’s observed narrative/narrator changes, which may be both helpful and harmful, which subsequently can improve or reduce the quality of life.

Narratives were accessed through six routes: the algorithmic recommender systems, random narratives, browsing pre-defined categories, engagement emails, and saved or highly rated hopeful narratives. After accessing a narrative, participants were asked to rate the narrative on hopefulness and, optionally, four other theoretically informed effects (connection to the narrator/narrative, empathy, and learning). Feedback ratings provided by participants contributed to the refinement of the recommender system. Engagement strategies and wellbeing features were embedded in the NEON Intervention. Engagement strategies included email and text message communication, gamification (receiving badges for narrative engagement), and a notes section at the end of each narrative. Safety features included the ‘I’m upset’ page providing information on coping (participants’ self-management strategies, general coping strategies, helplines/services) and a ‘Get me out of here’ function that immediately closes the intervention and redirects to a neutral webpage (google.com). Other NEON Intervention features are described elsewhere (10, 11).

### 2.4 Outcomes

Candidate primary and secondary outcome measures were evaluated. The Manchester Short Assessment of Quality of Life (MANSA), a 12-item measure of the quality of life with adequate psychometric properties (19), was scored on a 7-point rating scale (higher scores indicating higher quality of life); CORE-10, a 10-item measure of mental health distress [ranging from 0 (low distress) to 40] (20); abbreviated Herth Hope Index (HHI), a 12-item measure of hope [ranging from 4 (low hope) to 48] (21); the Mental Health Confidence Scale, a 16-item measure of self-efficacy in people with mental health problems [ranging from 16 (low empowerment) to 96] (22); and Meaning in Life Questionnaire (MLQ), a 10-item measure of the presence of meaning and the search for meaning in life [ranging 1 (low meaning presence/search) to 7] (23). The EQ-5D-5L was used to measure general health status. Using an English population dataset (24), the EQ-5D-5L profile data were converted into EQ-5D-3L utility values (UK tariff) using a mapping function parametrised on age and sex (25), as recommended by the National Institute for Health and Care Excellence (26).

### 2.5 Sample size

Power calculations are not required for feasibility trials. The rules of thumb for the overall sample size of a two-arm feasibility study are between 24 and 70 participants to estimate the standard deviation for a continuous outcome (27, 28). Conservatively, we aimed to recruit 100 carers (50 per arm).

### 2.6 Randomization and masking

Participants were individually randomized (ratio of 1:1) using permuted blocks of randomly varying lengths (2, 4, or 6). The randomization sequence was generated by an independent statistician at a trial unit. There was no stratification due to the feasibility of the trial. Participants self-enrolled for the trial via a website and were not blind to their allocation status.

### 2.7 Procedures

Trial recruitment began in March 2020 and was completed in March 2021, with the final follow-up completed in May 2022.

NEON trial consent procedures are described extensively elsewhere (10). Briefly, all recruitment messaging directed participants to the *splash page* for the NEON trials, which consisted of information about the trial, an avenue for safety reporting, and logging into the NEON Intervention. The trial information page provided access to a set of eligibility checking questions to ensure that individuals could take part in one of the three NEON trials without engaging in the full informed consent process.

Eligible individuals were presented with the participant information sheet and asked to indicate whether they wished to take part in the NEON trials. Participants provided informed consent via the web application used to deliver the NEON trials, where they had the option to provide consent to take part in the process evaluation. Participants were then able to create an account on the NEON Intervention and complete the baseline and demographic questionnaires. Repeat registrations by the same person were identified and suspended using a trial-specific procedure authorised by the TMG and PSC. Following the completion of the baseline questionnaire, participants were randomized into the intervention or control arm. Participants allocated to the intervention arm had immediate access to the intervention. Control group participants had access to a reduced version of the NEON Intervention home page, providing access to signposting and trial information. They were invited to use the intervention after completing the 52-week standardized measures. All participants continued with usual care.

Participants were invited to complete all outcome measures at baseline and at 52 weeks (primary clinical endpoint). The MANSA was also completed at weeks 1 and 12. All assessments were completed via the web application delivering the NEON trials, which generated automated messages to alert participants to complete the measures. Participants were reimbursed £20 for each set of outcome measures completed.

All participants who consented to take part in the qualitative process evaluation were contacted when they either had reached the primary endpoint and completed all outcome questionnaires or 32 days after the primary endpoint was reached (indicating data were late). Process evaluation interviews were conducted to evaluate the suitability and acceptability of the NEON Intervention and trial processes. Process evaluation interviews were guided by a topic guide (Supplementary material 1) and iteratively updated considering participant responses. The topic guide was piloted with two researchers and two carers of people with mental health problems. Interviews were conducted via telephone or video conferencing, which was subject to participant preferences. All interviews were audio−/video-recorded and pseudonymised to preserve anonymity. The interview length ranged between 22 and 60 min, and participants were reimbursed £20.

### 2.8 Analysis

All quantitative analyses were based on the intention-to-treat (ITT) sample (29).

For Objective 1 (trial parameters), the mean weekly recruitment rate and attrition rates were calculated using descriptive statistics. Power calculation to provide a preliminary sample size estimate for a definitive trial was done based on 90% power and attrition rates to determine the best-suited outcome measure. Candidate primary and secondary outcomes for a definitive trial were identified using effect size estimates (Cohen’s *d*). Effect sizes were calculated and compared between the intervention and control groups with their bootstrapped confidence intervals.

For Objective 2 (acceptability), a mixed-methods approach to understanding the rates of engagement was examined through descriptive statistics of the total number of logins, average number of sessions per week, average number of total and unique narrative requests, narrative feedback, and method of receiving stories. Randomization acceptability was evaluated as part of the qualitative process evaluation interviews.

For Objective 3 (feasibility), the qualitative process evaluation interviews were thematically analyzed, guided by a six-step process (30), to understand the suitability of the NEON Intervention for carers, particularly with trial processes and intervention content. First, interviews were transcribed verbatim, pseudonymised. Second, one researcher familiarized themselves with the data and developed a preliminary coding framework. Third, the preliminary coding framework was refined through discussion with the wider research team (*n* = 5). Fourth, two additional analysts double-coded 10% of transcripts to refine the coding framework and identify disagreements. Fifth, the revised coding framework was discussed with the wider research team to further refine the framework. Sixth, the revised coding framework was applied to all transcripts by the lead author.

## 3 Results

### 3.1 Participants

**Figure 1:**
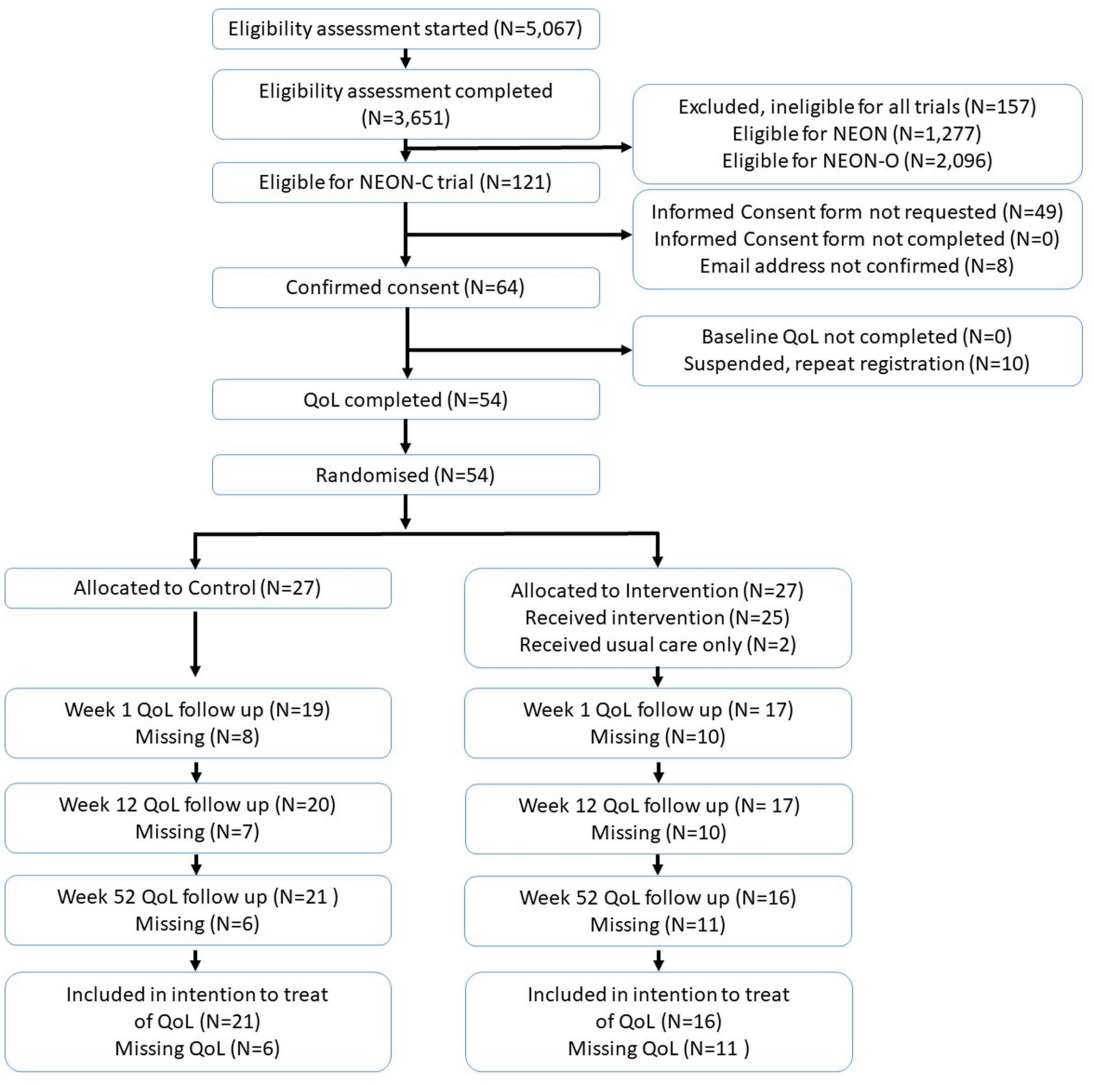
Trial consort diagram.

Between March 2020 and March 2021, a total of 5,067 individuals started eligibility screening for the NEON trials (including NEON and NEON-O), and 3,651 individuals completed the eligibility screening, with 121 of them eligible for the NEON-C trial (Figure 1). Consent was received from 64 participants. Ten participant accounts were suspended for repeat registration. Participants (*N* = 54) who completed the MANSA were randomized into intervention (*n* = 27) or control arms; therefore, this study did not recruit to target. Eleven participants were lost to follow up in the intervention arm and six in the control arm. A total of 36 participants were included in the ITT analysis. Baseline demographic characteristics for NEON-C participants are described in Table 1. Fourteen participants were interviewed as part of the process of evaluation.

**Table 1:** Baseline demographic characteristics.

Variable	Total (*N* = 54)	Intervention (*n* = 27)	Control (*n* = 27)
Gender: *n* (%)			
Woman	44 (81.5)	23 (85.2)	21 (77.8)
Man	8 (14.8)	≤5	6 (22.2)
Missing	≤5	≤5	≤5
Age at randomization, years: Mean (SD)	53.8 (11.1)	52 (11.4)	54.7 (11)
Ethnicity: *n* (%)			
White British	51 (94.4)	24 (88.9)	27 (100)
Other ethnicity	≤5	≤5	≤5
Missing	2 (3.7)	2 (7.4)	0 (0)
Region: *n* (%)			
East of England	≤5	≤5	≤5
London	≤5	≤5	≤5
Midlands	15 (27.8)	11 (40.7)	≤5
North East and Yorkshire	≤5	≤5	≤5
North West	≤5	≤5	≤5
South East	16 (29.6)	≤5	11 (40.7)
South West	6 (11.1)	≤5	≤5
Missing	≤5	≤5	≤5
Highest qualification: *n* (%)			
O-levels/GCSE/A-levels/AS-levels/NVQ or equivalent	12 (22.2)	6 (22.2)	6 (22.2)
Degree-level qualification	22 (40.7)	12 (44.4)	10 (37)
Higher degree level qualification	18 (33.3)	7 (25.9)	11 (40.7)
Missing	≤5	≤5	≤5
Occupation: *n* (%)			
Employed	34 (63)	17 (63)	17 (63)
Training and education	≤5	≤5	≤5
Unemployed	9 (16.7)	≤5	≤5
Retired	10 (18.5)	4 (14.8)	6 (22.2)
Living situation: *n* (%)			
Alone	≤5	≤5	≤5
With others	49 (90.7)	26 (96.3)	23 (85.2)
Ever used Primary Mental Health Services: *n* (%)			
Yes	36 (66.7)	16 (59.3)	20 (74.1)
Missing	≤5	≤5	≤5
Ever used Secondary Mental Health Services: *n* (%)			
Yes	13 (24.1)	7 (25.9)	6 (22.2)
Missing	≤5	≤5	≤5
Mental health problem in the last month: *n* (%)			
I did not experience mental health problems	39 (72.2)	19 (70.4)	20 (74.1)
I experienced mental health problems	13 (24.1)	6 (22.2)	7 (25.9)
Missing	≤5	≤5	≤5

### 3.2 Objective 1 (trial parameters)

A recruitment rate of 1.04 participants per week was achieved. Participants were recruited from seven mental health trusts in England (0.31 participants per week). No adverse events were reported.

Participants who were lost to follow-up affected all measures. For the MANSA, there were no missing values at baseline (*N* = 54), 33.3% attrition at 1 week, 31.5% attrition at 12 weeks, and 31.5% attrition at 52 weeks. Attrition rates in the control group decreased from 29.6% (1 week) to 22.2% (52 weeks). For all other measures, overall attrition was 5.6% at baseline and 35.2% at 52 weeks. Supplementary material 2 provides a breakdown of attrition rates between the intervention and control groups for all outcome measures.

Outcome scores at baseline and 52-week follow-up and the mean difference between the intervention and control groups at the 52-week follow-up are shown in Table 2. At the 52-week follow-up, participants in the intervention group reported a small effect on hope (*d* = 0.14), a moderate effect on the presence of meaning in life (*d* = 0.31), and a moderate effect on the search for meaning in life (*d* = 0.33). No significant changes were observed in the quality of life, distress, or self-efficacy. Control group participants reported higher health status at 52 weeks than intervention group participants, but this comparison does not account for the baseline imbalance in EQ-5D-3L utility values. Two candidate primary outcomes are recommended for consideration in a future definitive trial: (1) the Herth Hope Index and (2) the Meaning in Life presence sub-scale. For the Herth Hope Index, 3,305 participants would be needed (90% power, 0.05% significance, and 35% attrition) to power a full definitive trial. For the Meaning in Life questionnaire, 677 participants would be required to detect changes in the presence of the sub-scale.

**Table 2:** Outcome scores at baseline and the 52-week follow-up.

	Intervention			Control			Mean difference between intervention and control at 52-weeks	Effect size cohen’s *d* (bootstrapped CI)
	Baseline mean (SD)	52 weeks mean (SD)	Mean difference from baseline	Baseline mean (SD)	52 weeks mean (SD)	Mean difference from baseline		
Quality of Life (MANSA) (*N* = 37)	4.59 (0.82)	4.90 (0.68)	0.31	4.83 (0.83)	4.95 (0.73)	0.12	−0.05	−0.071 (−0.70 to 0.58)
Mental health distress (CORE-10) (*N* = 35)	12.52 (6.99)	10.50 (4.91)	−2.02	10.73(4.53)	10.57 (5.38)	−0.16	−0.07	−0.014 (−0.59 to 0.76)
Hope (HHI) (*N* = 35)	36.84 (5.19)	38.21 (4.06)	1.37	36.96 (5.75)	37.48 (5.92)	0.52	0.73	0.14 (−0.52 to 0.82)
Self-efficacy (MHCS) (*N* = 35)	67.92 (12.31)	72.00 (8.63)	4.08	69.85 (11.79)	71.48 (10.63)	1.63	0.52	0.053 (−0.59 to 0.76)
Meaning in Life (MLQ PRESENCE) (*N* = 35)	5.00 (1.29)	5.34 (0.95)	0.34	5.02 (1.25)	4.94 (1.45)	−0.08	0.40	0.31 (−0.39 to 0.89)
Meaning in Life (MLQ SEARCH) (*N* = 35)	4.02 (1.19)	3.86 (1.51)	−0.16	4.05 (1.44)	4.34 (1.43)	0.29	−0.48	−0.33 (−0.95 to 0.38)
EQ-5D-3L Median (IQR) (*N* = 35)	0.80 (0.73–0.93)	0.80 (0.74–0.89)	0.00	0.89 (0.80–0.89)	0.86 (0.78–0.89)	−0.03	−0.06	NA

### 3.3 Objective 2 (acceptability)

Participants logged into the NEON Intervention 258 times (average of 0.18 sessions per week). A total of 668 narratives were requested, representing 428 unique narratives. Participants accessed a mean of 25 narratives (SD = 78; range 0–407), with a median of 5 narratives (IQR = 2–11) in a 52-week period. Feedback ratings on the effect on hopefulness were provided for 523 (78.3%) narratives from 17 participants, with far fewer ratings (*n* = 15, 2%) for the other rating scales (connection to the narrator/narrative, empathy, and learning). The frequency for all narrative access routes is presented in Supplementary material 3. The main barrier to engaging with the NEON Intervention was reported as a lack of time and limited integration into participants’ daily lives. A minority of participants viewed their caring experiences as normal ups and downs of life, while they perceived the NEON Intervention as being more appropriate for people with mental health problems:

*‘In order to access the NEON Intervention, I would have to feel like something was an ongoing issue and it’s not. My mental health is just a normal fluctuation of life, that it could be fixed by having a good nap or having a chat to a friend. So that’s why it did not really suit me’* (Juliet).

Simple layout and navigation ease facilitated the use of the NEON Intervention. Some participants reported difficulty reading narratives on a smartphone and digital exclusion.

*‘Some people will not have technology available to them…. I found it quite difficult reading the stories on my telephone…. Older people would struggle to be able to have access’* (Kate).

Some carers did not benefit from engagement features due to not noticing them or perceiving them as ineffective.

*‘I do not want touches. I really do not feel the need or the benefit of that [engagement features] …’* (Mira).

Some participants expressed disappointment when randomized into the control group, and randomization was deemed unacceptable if they signed up for the NEON Intervention when starting their caring journey or during times of crisis. Overall, randomization acceptability was influenced by participant circumstances at the time of randomization.

*‘If people had to wait a long time to get access to stories…, can they afford to wait?’* (Kerrie).

No participant raised concerns about the NEON Intervention sign-up procedures or the communication frequency or style of the research team. One participant wanted clarification on how frequently they were expected to use the intervention.

### 3.4 Objective 3 (feasibility)

The concept of the NEON Intervention was considered suitable for use by informal carers. However, participants recommended five modifications to enhance the user experience, increase applicability to carers, and reduce confusion in trial processes.

First, the inclusion of carer perspective narratives was recommended by most participants (*n* = 12). Carer perspective narratives were reported as being potentially beneficial through enhancing relatability to narratives, providing strategies for coping with or overcoming situations, and supporting a sense of personal identity greater than their carer identity. For some participants, lived experience narratives did allow them to *‘develop understanding, empathy and perspective’* (Charlie). Most participants reported that they saw benefits to having both lived experience and carer perspective narratives within the intervention. Two specific types of narrative were recommended: information/resource-related and feelings/experience-related. Participants reported a greater need for information during periods of crisis. Examples of information/resource narratives included narratives from the carer perspective on managing situations, diagnostic information, and communicating with health professionals. Feeling/experience-related narratives to support carer wellbeing were also recommended, particularly during non-crisis periods, to assist with retaining personal identity beyond being a carer.

*‘Ensuring that I retain my integrity… I’m an individual in my own right and I do not get drawn down by episodes which aren’t me’* (Stacy).

*‘It was me as a carer. I was the one that was in need. My [cared for person] had the most amazing support and intervention… I was left having to navigate it. So absolutely, it’s the carer perspective and that there is hope for the carer as well as for the cared for’* (Steph).

Second, signposting to carer support services and information as part of the NEON Intervention was recommended by four participants (33%). This recommendation was exemplified by a carer’s need for information during the early stages of the caring journey and periods of crisis. Examples included information about local carer support groups, national services, and carer services within mental health services.

*‘[Carers] need a lot of signposts and information, whether through phone calls, regular contact, booklets, leaflets about where to go, who to contact during emergencies or questions they need to ask. For a new carer, it can be a whole range of rules.’* (Gemma).

Third, while the NEON Intervention was considered easy to navigate, and no concerns were raised about the sign-up procedures or the communication frequency/style of the research team. Four participants suggested modifications to enhance the user experience. These modifications included the ability to indicate that they are a carer within the NEON Intervention, wording to highlight which perspective questions are being framed from (carer or lived experience perspective), clarifications of usage expectations, keyword searching within narratives, country-specific narratives to maximize relevance and connection, and accessibility through a standalone mobile application. Outcome measure completion was difficult for four participants such that the perspective in which questionnaires should be completed (carer vs. cared for perspective) was confusing.

*‘I struggled with answering a lot of the questions on the surveys because it wasn’t geared at being a carer. It was geared at having the illness’* (Miriam).

Fourth, developing an interactive community was suggested (*n* = 3). Examples include communicating with other participants via online forums that mimic in-person support, group messaging, writing their personal stories, or leaving comments for narrators. Other participants reported that only receiving narratives from the carer’s perspective would be sufficient to create a supportive community.

*‘Group messaging to two individuals who are going through similar experiences might be helpful’* (Kate).

*‘If there was some interactive chat… I wanted to write my own story… in response to one of the stories, I did not notice an option to do that… Maybe also reading the story you could leave comments and have some bigger discussion about what it brought up for you or some other people’s experience or reactions to it’* (Mel).

Fifth, the privacy needs of carers require consideration. Two participants acknowledged the need for ‘authentic’ narratives to promote connection between carers, which may be facilitated through referencing specific experiences within narratives. However, it is important to be able to identify individuals with narratives and the potential negative impact (e.g., feelings of burden) this identification may have if people they care for recognize themselves within narratives (see Supplementary material 4 for additional quotes).

*‘I can have this frank conversation with you, but if I had it with my [partner] in the room, I would not be saying the same things because… not for a second do I want them to think that is a burden.’* (Juliet).

## 4 Discussion

This first-in-field study investigated the feasibility and acceptability of recorded mental health recovery narratives for carers of people with mental health problems. Objective 1 identified that it is feasible to recruit carers into a trial, and candidate outcomes for a definitive trial include hope and the presence of meaning in life; however, attrition rates were high. Objective 2 identified that engagement in the intervention was wide-ranging; however, as a whole, participants engaged in, on average, 25 narratives over 52 weeks. Objective 3 identified five recommendations to enhance the applicability, user experience, and trial processes of the NEON Intervention.

While it was feasible to recruit carers into the NEON Intervention, the trial did not recruit to target, which could be explained through the narrow inclusion criteria. Mental health carers have higher rates of mental health problems than those without caring roles (31). Given that only carers who had not experienced mental health distress in the past 6 months were eligible for NEON-C, it is plausible that a greater number of carers who started the eligibility screening process yet were allocated to the NEON or NEON-O trials due to personal experiences of mental health problems. It is possible that the recruitment rate achieved may be an underestimation of the possible recruitment rate when including carers with mental health problems. Amending the eligibility criteria to include carers with mental health concerns would allow for an increased sample size, allowing for better representation of the carer population and a powered trial.

The high attrition rates in the study reflected those of other trials of digital interventions (4, 5). Interestingly, the attrition rates in the control group improved over time, and more control group participants took part in the process evaluation. There are three possible reasons: (1) control group participant’s desire to use the NEON Intervention, (2) some aspects of the intervention influence attrition rates (e.g., additional time burden for already busy carers), and (3) amendment of trial processes so that all participants were reimbursed £20 for completing the MANSA at the primary endpoint.

Low engagement in the intervention was a point of concern. There may be three explanations for this. First, the relevance of an intervention to the end user may influence participant engagement. The NEON Intervention and its subsequent trial processes were designed for people with mental health problems in mind, not carers. The five recommendations identified in the process evaluation may serve as a starting point for adaptation. The involvement of carers and other stakeholders in the refinement of theory, intervention adaptations, and trial processes is strongly recommended to ensure a relevant intervention and robust trial. Ensuring that trial processes are geared toward carers is particularly important as four participants reported confusion over which perspective to answer outcome measures from (carer vs. cared for). Additional feasibility testing of adaptations is recommended prior to any definitive trial evaluation. Second, there was neither dosage requirement in this intervention, nor adherence measured. While other definitive trials of digital interventions for carers have also reported low or not reaching the minimal dosage requirement (4), based on current findings, it is impossible to ascertain what dose/level of engagement is required to be of benefit. While further feasibility testing could delineate this, it is acknowledged that there needs to be a balance between the demands placed on carers to participate and the potential therapeutic dose. Third, the stage of caring was identified as a factor influencing engagement. Early caring stages were associated with a need for information on where and how to seek help and coping strategies, which was consistent with the finding that randomization to the control group is less acceptable at an earlier stage of caring or during crisis periods and highlights the different needs of carers across the caring journey. While there is limited understanding of how the stage of recovery affects the caring journey, participants reported the need for a balance of narratives depicting experiences of seeking support and information and supporting a carer’s emotional wellbeing. Future trials may benefit by stratifying randomization based on the stage of care or the current experience of crisis. Moreover, this highlights the significant gaps in demographic characteristics collected about our carer participants (e.g., hours spent caring per week, who they care for, and caring stage).

Engaging in the NEON Intervention was found to have a small effect on hope and a moderate effect on the presence of meaning in life in carers. These may be the candidate primary outcomes for a future trial. The sample size calculation indicates that 3,305 participants are required to detect an effect for the HHI and 667 participants for the MLQ. Given that this trial did not recruit enough participants, it is unknown whether it is possible to recruit the required number of participants to detect an effect for either the HHI or MLQ. Other digital interventions targeted at carers of people with mental health problems have previously enrolled 400–800 carers in a randomized trial; therefore, it is conceivable that it may be possible to power a trial when using the MLQ as the primary outcome. This may further the case by amending the eligibility criteria to include carers who have personal experience with mental health problems.

Overall, this feasibility study identified that the NEON Intervention is feasible and acceptable to carers with no current personal mental health problems in the trial. There are small to moderate effects on hope and the presence of meaning in life. Significant adaptation of the intervention and its theory base for carers is required to ensure relevance. Additional feasibility testing is recommended prior to a definitive trial.

## Data availability statement

Anonymous and pseudonymous elements of the datasets used and/or analysed during the study will be available on reasonable request from the study team before the NEON study ends.

## Ethics statement

The studies involving humans were approved by East Midlands Leicester REC 19/EM/0326. The studies were conducted in accordance with the local legislation and institutional requirements. The ethics committee/institutional review board waived the requirement of written informed consent for participation from the participants or the participants' legal guardians/next of kin because online informed e-consent was provided via the web-application that delivered the NEON Intervention.

## Author contributions

FN: Conceptualization, Data curation, Formal analysis, Investigation, Methodology, Project administration, Writing – original draft, Writing – review & editing. SR-E: Conceptualization, Data curation, Investigation, Methodology, Project administration, Supervision, Writing – review & editing. JO: Writing – review & editing. CN: Conceptualization, Data curation, Formal analysis, Investigation, Methodology, Supervision, Writing – review & editing. JL-B: Conceptualization, Data curation, Formal analysis, Investigation, Methodology, Writing – review & editing. CY: Writing – review & editing. YA: Data curation, Writing – review & editing. KP: Formal analysis, Methodology, Writing – review & editing. YK: Writing – review & editing. SP: Writing – review & editing. SPG: Formal analysis, Writing – review & editing. LK: Writing – review & editing. DR: Writing – review & editing. SG: Writing – review & editing. GT: Writing – review & editing. MS: Conceptualization, Data curation, Formal analysis, Funding acquisition, Investigation, Methodology, Project administration, Writing – review & editing.
