# Supplementary material for: Pragmatic, feasibility randomized controlled trial of a recorded mental health recovery narrative intervention: narrative experiences online intervention for informal carers (NEON-C)
Source: Front Psychiatry. 2024 Jan 23;14:1272396. doi: 10.3389/fpsyt.2023.1272396 (PMC10845336; doi:10.3389/fpsyt.2023.1272396)
Supplement: Supplementary file 1 [file Data_Sheet_1.docx]

**Online Supplement 1 Topic Guide**

1. Could I get a sense of who you care for or support with their mental health, what is your relationship like, and how much of your time this takes up each week?

2. What were your motivations for using the NEON website?

*Prompts:*

- - Can you remember how you found out about the NEON website, and why it of interest to you?
  - Scoping out NEON for the person they cared for (not long term usage)

3. What did you hope/expect to get out of using the NEON intervention?

4. Did the person that you care for/support know that you are using the NEON intervention?

4.a. Have you ever shared or discussed any of the stories with the person you care for/support?

5. Were the stories on the NEON website relevant to you?

*Prompts:*

- Were the stories on the NEON website what you had expected?
- What kinds of stories were you looking for? (e.g. for own mental health, to support them in caring for someone, to gain a deeper understanding of the person they care for?)
- Did you discuss the stories you saw on the NEON website with the person you care for? What was their reaction to this?
- Has the stories had a positive or negative effect on you or your relationship with the person you care for?
- We only have stories from people living with mental health issues. Are there any ways that also having stories about carers or supporters would have been helpful to you? If yes, can you elaborate?
- What changes would you make (in any) to make NEON better for carers or supporters?

6. Were there any aspects of the NEON website that facilitated your use of the intervention?

*Prompts:*

- Website in general (layout, aesthetics, navigation)
- Features (matching, random story, browse, self-management strategies, get me out of here, engagement badges, notes)
- Communication from the NEON team (frequency, method)

7. Were there any barriers which prevented you from using the NEON website?

*Prompts:*

- Distress experienced as a result of NEON (how did you manage your distress?)
- Other

8. As part of the sign up procedures, you could receive stories from the NEON website immediately, or wait one year before getting access. Would having to wait for a year make you less likely wanting to take part?

9. Control participants only: Was waiting for a one year before being able to access stories on the NEON website acceptable?

10. Thinking back to the signing up process for the intervention, were there any part that was difficult?

11. What was it like to complete the outcome measures? (these are the surveys we have been asking you to fill in every few months)

11.a. *Prompt:* frequency*, number of questions*
