# Supplementary material for: Pragmatic, feasibility randomized controlled trial of a recorded mental health recovery narrative intervention: narrative experiences online intervention for informal carers (NEON-C)
Source: Front Psychiatry. 2024 Jan 23;14:1272396. doi: 10.3389/fpsyt.2023.1272396 (PMC10845336; doi:10.3389/fpsyt.2023.1272396)
Supplement: Supplementary file 2 [file Data_Sheet_2.docx]

**Online Supplement 2. Attrition rates for outcome measures across follow-up periods**

|  | **Intervention Group**  **(n=27)** | **Control Group**  **(n=27)** | **Total Attrition Rate**  **(N=54)** |
| --- | --- | --- | --- |
| **MANSA** | | | |
| Baseline  (N=54) | 0  (0%) | 0  (0%) | 0  (0%) |
| 1 Week  (N=36) | 10  (37.0%) | 8  (29.6%) | 18  (33.3%) |
| 12 Week  (N=37) | 10  (37.0%) | 7  (25.9%) | 17  (31.5%) |
| 52 Week  (N=37) | 11  (40.1%) | 6  (22.2%) | 17  (31.5%) |
| **All Other Outcomes** | | | |
| Baseline  (N=51) | 2  (7.4%) | 1  (3.7%) | 3  (5.6%) |
| 52 Week  (N=35) | 13  (48.1%) | 6  (22.2%) | 19  (35.2%) |
