# Supplementary material for: Pragmatic, feasibility randomized controlled trial of a recorded mental health recovery narrative intervention: narrative experiences online intervention for informal carers (NEON-C)
Source: Front Psychiatry. 2024 Jan 23;14:1272396. doi: 10.3389/fpsyt.2023.1272396 (PMC10845336; doi:10.3389/fpsyt.2023.1272396)
Supplement: Supplementary file 3 [file Data_Sheet_3.docx]

**Online Supplement 3. Frequency of all narrative access routes**

| **Narrative Access Route** | **Frequency** |
| --- | --- |
| Random narrative | 385 |
| Algorithmic recommender system | 170 |
| Browse narrative categories | 71 |
| First narrative | 25 |
| Narratives rated as hopeful | 8 |
| Narratives received via email | 7 |
| Narratives marked as favourites | 2 |
