# Supplementary material for: Pragmatic, feasibility randomized controlled trial of a recorded mental health recovery narrative intervention: narrative experiences online intervention for informal carers (NEON-C)
Source: Front Psychiatry. 2024 Jan 23;14:1272396. doi: 10.3389/fpsyt.2023.1272396 (PMC10845336; doi:10.3389/fpsyt.2023.1272396)
Supplement: Supplementary file 4 [file Data_Sheet_4.docx]

**Online Supplement 4. Themes and Quotes Arising from the NEON-C Process Evaluation**

| **Objective 2 (Acceptability)** | |
| --- | --- |
| Barriers | ‘I think I just forgot… maybe I could have done with more reminders’ (Mel)  ‘No, I did suggest to my sister that she might want to join… She didn’t… but remember her saying to me that ‘I’ve looked at it and it’s not right for me’’ (Kate)  ‘[there were times where I could read stories] but my most recent crash blip [was a barrier]’ (Mira)  ‘Although it’s kind of good to know that they’re there, I haven’t used NEON as much as I thought I would. I do think it’s a really good idea and I’m interested in the idea of recovery stories. I don’t know why I haven’t used it more. I did start a new job and it’s been quite full on and I’m working with other carers now’ (Mel)  ‘I can’t think of any barriers, anything that prevented me other than, you know just normal day to day stuff and you just get on. Unfortunately, and this is no ones fault, it is simply where we are, not least because of the pandemic which has had one of the huge benefits of pushing digital awareness functionality way quicker than we would have achieved without it and I am guilty of this as much as anybody else is we are flooded with information’ (Stacy) |
| Engagement features | ‘I’ve got to be honest, I wasn’t aware of that’ (Matthew)  ‘No, I’m afraid not, but don’t take anything from that, it could be just that you know, I’ve been there and you know...don’t do those sort of things but actually it isn’t because I want to learn and be able to be more effective’ (Stacy) |
| Randomisation | ‘I suppose because I had other avenues for support and insight, it wasn’t a massive issue for me. But perhaps having had access earlier it’s bound to enhance your skills and insights. If people had to wait a long time to get access to stories… can they afford to wait? You know… if these stories are going to support and help support holistic wellbeing, perhaps sometimes sooner the better. I read a book recently written by the parents of a young man who died, and it was called ‘He died waiting’ and when you said about waiting a year, it just reminded me of that’ (Kerrie)  ‘I got this straight away, I would be so cross if I’d had to wait. Because I think you know at the point of interest, you’ve captured my attention. I’m interested, I’m now committed. To make me wait would just be laughing’ (Charlie)  ‘I suppose [it was] just somewhat frustrating really. As I say, [it] reinforced this feeling of I’m not actually contributing a lot to this because I can’t answer various questions that probably would benefit from a worthwhile answer’ (Miriam) |
| Sign Up Procedures | ‘I don’t think it was difficult. It’s always, you know when something is new and [you are] never quite sure how it’s going to work. It was ok as far as I can remember’ (Cathy)  ‘No because once [partner’s name] set it up for me, it was straight forward from then (Gemma)  ‘I can’t recall anything that put me off or was difficult or confused me. I can’t think of anything at all’ (Stacy) |
| Communication | ‘I get regular emails, it kind of reminds me that the site is there and I go on. If it wasn’t for those, I might tend to forget about it, but because wen they come I always have a quick look around’ (Cathy)  ‘To be honest, I just ignored that email after awhile. But the text messages, it was like ‘oh it’s a real person’… where as with the email it is a bit like ‘well that’s just automated’ I’m just going to ignore that’ (Juliet)  ‘That was a good reminder that it’s still going on’ (Mel)  ‘I actually don’t remember receiving them every month and I think that every time I had received them it sort of went ‘oh yeah ok I must go and do something with that’. I think that had I received them every month I would have found it a bit tiring. Maybe there is something about the starting time (initial period) when you’re getting somebody recruited onto the site and finding the use of it, is then a couple of reminders and thereafter drifting off so that you can, if you like, depend on a person learning to access that site and the value from it. And I suppose also because I’m pretty much aware of what’s going on, I haven’t felt the need for that sort of support. Having said that, I think if this would have been available years ago, I think I might have been on it most days’ (Stacy) |
| **Objective 3 (Feasibility)** | |
| Narratives from the Carer Perspective | ‘If you want to aim at carers, then it would be good if they included like some things where they found solutions like for example one of the things I had was that she because she had different carers as every day’ (Cathy)  ‘It was me as a carer. I was the one in need. My [person they care for] had the most amazing support and intervention. It was me as a carer that was left having to navigate it myself. So yeah, absolutely, it’s the carer perspective and that there is hope for the carer as well as for the cared for’ (Steph)  ‘I think hearing the stories from the mental health users perspective was valuable.’ (Gemma)  ‘I would like to be able to access both. I think if there would be an option to you know say do you want a service user story or a carer story… but to be able to opt into both’ (Mel) |
| Signposting to Services | ‘To a certain extent something on the lines of a local carers groups.  I volunteered with the carers support group… It is information, it is education and it is also listening’ (Gemma)  ‘In terms of for example, preparing carers for the information is maybe letting them know if you’re aware of what it is that they’re concerned with, are there new guides, supports, pieces of information, experiences so maybe just updating and letting people just know, you know, check on because there is a new piece of information that might be helpful for you.’ (Stacy)  ‘Where to go for help… to make carers aware that there are organisations in their area’ (Mel) |
| Interface Modifications | ‘In the profile page, to allow a section where you can identify yourself as a carer… I think just the need to acknowledge that although you’re not the one with the illness’ (Miriam)  ‘I don’t know if there’s any kind of search facility, so when I went on I had to click on different categories like female or psychosis. There were various things I could click on, but I didn’t want to, I wanted to type something in a search box’ (Steph)  ‘Maybe some people will not have technology available to them. I’m quite fortunate, I have a laptop. I found it quite difficult reading the stories on my telephone. Young people might be [better able to read on their phone], but I guess older people would struggle to be able to access’ (Kate)  ‘From the outset, I struggled with answering a lot of the questions on the surveys because it wasn’t geared at being a carer. It was geared at having the illness’ (Miriam)  ‘I was a bit confused as to why I was filling it in and what was the purpose behind it’ (Matthew)  ‘When I’ve been asked questions or if I’ve had to enter any information, it hasn’t really differentiated between myself and the person I care for. I think you are asking me, but I’m not sure really…’ (Mel) |
| Interactive Community | ‘I wanted to write my own story… I wanted to sort of say this was my experience, say in response to one of the stories. I didn’t notice that there was an option to do that’ (Mel)  ‘I can see that for some people that more one to one contact or maybe group messaging of two individuals who are going through similar experiences might be helpful’ (Kate) |
| Privacy Considerations | ‘I do some training for the mental health trust, and I do carers training for them and for some of their staff. They have asked for video bits and pieces, and we have always said, that’s absolutely fine, providing that it’s only staff who can ever see this. I don’t want the things that I talk about to be seen by my folks, my wife, or for any of her friends to end up seeing it. Because it’s personal. I am prepared to share my knowledge and what I’m going through in order for better services. In order for that to happen, I have to be honest as humanly possible. Otherwise you’re not getting the true story and you’re not going to alter your practice in order to be able to try and help people. But actually, if I said some of these things to her that could be very detrimental. I think that would be my only concern.’ (Matthew)  ‘This is a tricky one. Because I know I could have this frank conversation with you, but if I had it with my husband in the room. I wouldn’t be saying the same things, because noy for a second do I want him to think that is a burden, I could say is a pain in the ****, but equally I’m annoying to him.’ (Juliet).  ‘People spoke about their difficulties and challenges in a very honest and open way. It didn’t feel at all scripted or contrived. It just felt brutally honest’ (Charlie) |
